# Lung Cancer Management: Revolutionizing Patient Outcomes Through Machine Learning and Artificial Intelligence

**DOI:** 10.1002/cnr2.70240

**Published:** 2025-07-17

**Authors:** Taghi Riahi, Bahareh Shateri‐Amiri, Amirhossein Hajialiasgary Najafabadi, Sina Garazhian, Hanieh Radkhah, Diar Zooravar, Sahar Mansouri, Roya Aghazadeh, Mohammadreza Bordbar, Shirin Raiszadeh

**Affiliations:** ^1^ Department of Internal Medicine, School of Medicine, Rasool Akram Medical Complex Iran University of Medical Sciences Tehran Iran; ^2^ Research Group Quantitative and Computational Biology, Max‐Planck‐Institute for Multidisciplinary Sciences University of Göttingen Göttingen Germany; ^3^ Research Group of Quantitative and Systems Biology, Max‐Planck‐Institute for Multidisciplinary Sciences (MPI‐NAT) University of Göttingen Göttingen Germany; ^4^ Department of Internal Medicine, School of Medicine, Sina Hospital Tehran University of Medical Sciences Tehran Iran; ^5^ School of Medicine Iran University of Medical Sciences Tehran Iran

**Keywords:** classification metrics, CT scan, deep learning, lung cancer, machine learning, ResNet50, transfer learning, tumor segmentation

## Abstract

**Background and Aims:**

Lung cancer remains a leading cause of cancer‐related deaths worldwide, with early detection critical for improving prognosis. Traditional machine learning (ML) models have shown limited generalizability in clinical settings. This study proposes a deep learning‐based approach using transfer learning to accurately segment lung tumor regions from CT scans and classify images as cancerous or noncancerous, aiming to overcome the limitations of conventional ML models.

**Methods:**

We developed a two‐stage model utilizing a ResNet50 backbone within a U‐Net architecture for lesion segmentation, followed by a multi‐layer perceptron (MLP) for binary classification. The model was trained on publicly available CT scan datasets and evaluated on an independent clinical dataset from Hazrat Rasool Hospital, Iran. Training employed binary cross‐entropy and Dice loss functions. Data augmentation, dropout, and regularization were used to enhance model generalizability and prevent overfitting.

**Results:**

The model achieved 94% accuracy on the real‐world clinical test set. Evaluation metrics, including *F*1 score, Matthews correlation coefficient (MCC), Cohen's kappa, and Dice index, confirmed the model's robustness and diagnostic reliability. In comparison, traditional ML models performed poorly on external test data despite high training accuracy, highlighting a significant generalization gap.

**Conclusion:**

This research presents a reliable deep learning framework for lung cancer detection that outperforms traditional ML approaches on external validation. The results demonstrate its potential for clinical deployment. Future work will focus on prospective validation, interpretability techniques, and integration into hospital workflows to support real‐time decision making and regulatory compliance.

**Abbreviations**
AI: artificial intelligence
CNN: convolutional neural network
CT: computed tomography
ML: machine learning
MLP: multi‐layer perceptron
ROC: receiver operating characteristic

## 1 Introduction

Lung cancer remains one of the most pressing global health challenges, ranking among the leading causes of cancer‐related mortality worldwide. Early and accurate detection of this disease is crucial for improving patient prognosis, and recent advancements in the fields of machine learning (ML) and artificial intelligence (AI) have showcased immense potential in transforming the management of lung cancer [1, 2].

Researchers have leveraged the power of deep learning algorithms to detect lung cancer in its earliest stages, utilizing chest radiographs and electronic medical record data [3, 4]. These innovative AI‐based models have demonstrated remarkable accuracy in predicting individual risk of lung cancer, enabling earlier diagnoses and, consequently, superior outcomes for patients [5].

Building upon these early detection capabilities, the integration of ML techniques with medical imaging, such as computed tomography (CT) scans, has facilitated more precise diagnosis, personalized treatment planning, and enhanced prognostic assessment for lung cancer patients [4, 6]. These data‐driven approaches hold the promise of streamlining clinical decision‐making processes and empowering healthcare providers to deliver truly personalized, evidence‐based care [1].

The synergistic implementation of ML and AI is revolutionizing the landscape of lung cancer management, from the earliest stages of disease detection to the optimization of targeted therapies and prognostic prediction [2, 5]. These transformative advancements are not only enhancing patient outcomes but also equipping clinicians with the tools to make more informed treatment decisions and propelling researchers to push the boundaries of lung cancer research.

Among the various deep learning techniques, transfer learning has emerged as a prominent approach in image recognition and computer vision [7]. This method leverages the knowledge gained from solving one problem to address a related, yet distinct, task. Typically, this knowledge is derived from pretrained models that have been trained on extensive datasets. In the realm of image‐based tasks, several well‐established pretrained models, such as ResNet [8], have proven to be highly effective. ResNet, in particular, has been extensively utilized for tumor recognition and classification in various medical domains, including lung cancer [9, 10, 11], glioblastoma [12, 13], and other diseases [10].

The success of these applications has spurred ongoing research to fine‐tune and develop new iterations of these models, tailored to specific tasks and datasets. One critical application is the early diagnosis and prediction of lung tumor regions from CT‐scan images. Given the complexity of CT‐scan images, accurately identifying tumor regions can be challenging. In this context, neural networks offer significant potential to assist clinicians in more accurately locating and evaluating tumor regions, thereby enhancing the diagnostic process.

This comprehensive research study aims to further explore and expand the potential of ML and AI in improving lung cancer prognosis, therapy response, and overall survival rates. By incorporating rich clinical and genomic data into innovative predictive models, the researchers seek to elevate patient care, facilitate evidence‐based treatment decisions, and ultimately make significant strides in the global fight against this devastating disease.

## 2 Methods

### 2.1 Study Design

In this study, our objective was to develop a neural network model using transfer learning to accurately predict tumor regions in CT‐scan images. To achieve efficient feature extraction and precise segmentation, we leveraged the power of the pretrained ResNet50 model [14]. We considered two learning tasks to first fine‐tune ResNet50 for tumor location reconstruction and then (cancer vs. normal) classification. Using the first task, we fine‐tuned ResNet50 parameters and used the refined parameters for the classification task.

In this setting, an encoder–decoder, also called a U‐net architecture, was designed using ResNet parameters for the encoder part. This allowed us to design a task‐specific image segmentation algorithm out of ResNet. The fine‐tuned encoder's extracted features were fed to a multi‐layer perceptron (MLP) model to perform the classification task. Both settings were performed on different datasets. By separating the feature extraction and classification stages, we ensured that the model could effectively capture the intricate patterns associated with lung cancer in CT‐scan images.

To evaluate the robustness and generalizability of our approach, we tested the model's performance on a real‐world patient dataset. This dataset, which had not been used during the training phase, provided a critical benchmark to assess the model's effectiveness in a clinical context. The results of this testing phase were crucial in determining the model's practical applicability and its potential for deployment in real‐world medical scenarios.

### 2.2 Data Collection

The primary data for this study was sourced from four distinct repositories to ensure a diverse and comprehensive dataset. The first two sources were well‐established chest CT‐scan datasets, both publicly available on Kaggle. These datasets are widely recognized and utilized in the research community for lung cancer studies. For the reconstruction task, we used an available CT nodule/lesion dataset https://www.kaggle.com/datasets/piyushsamant11/pidata‐new‐names/data. This dataset consists of 2535 lung CT scan images, not only from cancer patients but also from other diseases like COVID‐19 and infectious diseases. The annotation files provided the position of the nodules/lesions of the CT scan images. For the classification task, we used a hybrid dataset of https://www.kaggle.com/datasets/mohamedhanyyy/chest‐ctscan‐images and https://www.kaggle.com/datasets/hamdallak/the‐iqothnccd‐lung‐cancer‐dataset. These datasets contain only lung cancer and normal CT scan images. We meticulously combined images representing various types of lung cancer to construct a robust dataset, comprising 1346 images of cancerous lungs and 506 images from patients without lung cancer. This consolidated dataset was instrumental in the training and validation of our deep learning model, providing a balanced representation of both cancerous and noncancerous cases.

For the testing phase, we incorporated a third dataset, consisting of CT‐scan data from 72 lung cancer patients at Hazrat Rasool Hospital, a prominent medical institution in Iran. This dataset was exclusively used to evaluate the model's performance in a clinical setting, ensuring its applicability to real‐world scenarios. The inclusion of this hospital dataset was crucial in validating the model's effectiveness on data not seen during the training phase, thereby testing its generalizability and robustness.

### 2.3 Model Architecture and Training

In this study, we utilized the ResNet50 model as the initial model for our experiments. We had two distinct training settings.

In the first setting, we designed a reconstruction/segmentation task aimed at fine‐tuning the ResNet parameters to identify the tumor/lesion locations on CT images. We began by removing the final layer of the ResNet50 model and then added 5 up‐sampling layers along with 11 convolutional layers to reconstruct the original input image dimensions. This additional structure acts as a decoder, with the ResNet serving as the encoder. The entire architecture resembles a U‐Net model. The model was trained to reconstruct only the tumor/lesion positions from the CT scan data. In this setting, the ResNet model parameters were set to trainable mode. We used the Adam optimizer with a very small learning rate (1e‐8) to ensure that the learned parameters in the original ResNet50 were not significantly altered. The training was conducted over 30 epochs, resulting in a smooth training process. We found that using a combination of binary cross‐entropy and Dice loss functions provided more stable and generalizable learning outcomes.

The second learning task was built upon the trained ResNet‐based U‐Net from the first task. In this setting, we utilized only the encoder's extracted features. After flattening these features, we fed them into a two‐layer MLP model with 512 and 256 neurons in each layer, respectively. We applied the rectified linear unit activation function and a dropout rate of 0.3 for each layer. The objective of this task was to perform binary classification between cancerous and normal CT scan images. In this setting, the encoder parameters were frozen, and only the MLP parameters were trained.

In both settings, we used a training and validation dataset split ratio of 0.85 to 0.15. Each setting was trained on different datasets to ensure the robustness and generalizability of the model.

To prevent overfitting in both tasks, we incorporated dropout layers and augmentation techniques. We chose rotation, width and height shifts, shearing, zooming, and horizontal flipping methods to perform augmentation. Additionally, selecting optimal loss functions, optimizers, learning rates, and batch sizes contributed significantly to avoiding overfitting. Furthermore, during the training process, we selected the models with the lowest validation loss as our optimal models.

### 2.4 ML Model Benchmark

To see whether AI and deep learning can achieve higher performance compared to traditional ML methods, we evaluated the performance of support vector machine (SVM), XGBoost classifier, naïve Bayes classifier (NBC), and K nearest neighbor (KNN) classifier. The classifiers were implemented using the default hyperparameters provided by the scikit‐learn library (version 1.6.1). No additional hyperparameter optimization or tuning was performed. The model was trained on the original training dataset and subsequently evaluated on an independent test set. Prior to training and testing, all features were normalized/standardized, if applicable.

### 2.5 Inference Time and Computation Resources

The models were trained using the NVIDIA T4 Tensor Core GPU. During inference, the U‐Net part performs each prediction in 23 ms of GPU time, and the classification model performs in 2.5 ms per CT‐scan image.

## 3 Results

### 3.1 Model Performance on Lesion Region Recognition

**FIGURE 1:**
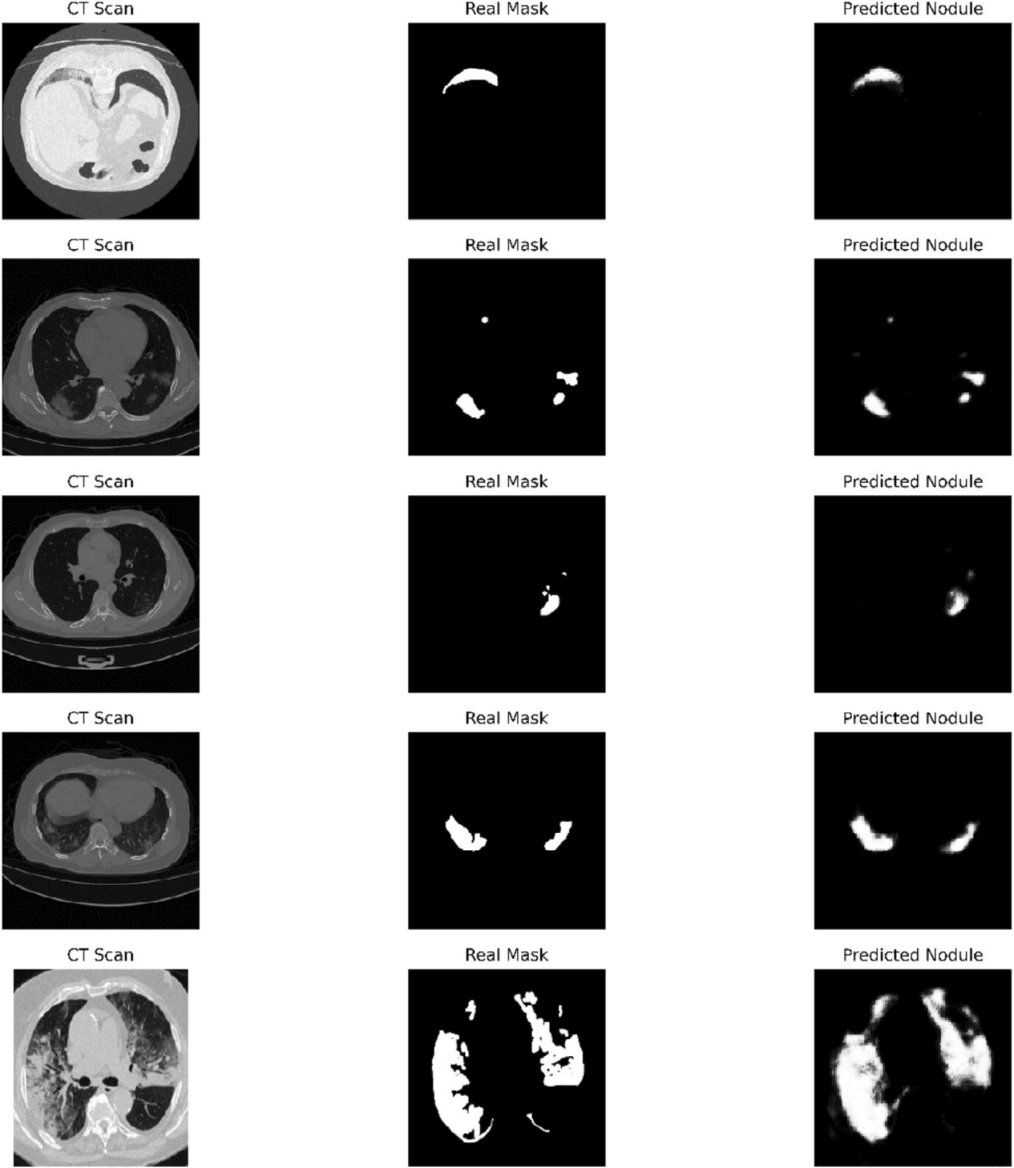
The model lesion recognition performance. The model's effectiveness in reconstructing tumor regions from CT scans.

In our first training task, which involved reconstructing the tumor regions from the original CT scan images, we used a ResNet architecture as the encoder, paired with a randomly initialized decoder. The model was fine‐tuned initially using binary cross‐entropy loss and subsequently with Dice loss. In this setting, the model was trained on a broader range of images that included not only lung tumors but also lesions and nodules. The model achieved a best BCE loss of 0.3 and Dice loss of 0.34 in the validation set. The model's performance is visualized on five test samples in Figure 1. Performance metrics for all testing data are provided in Supporting Information.

### 3.2 Model Performance on Binary Classification Task

**FIGURE 2:**
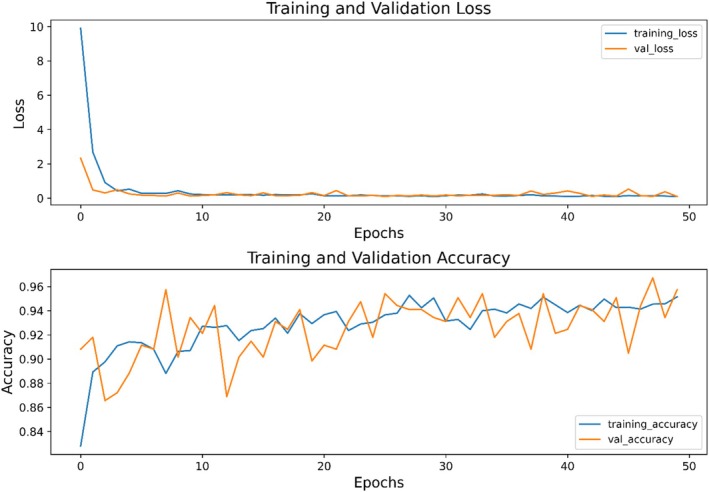
Training progress on the classification task. The training accuracy and loss metrics over epochs for the binary classification task, highlighting the model's performance in distinguishing cancerous from normal CT scans.

**FIGURE 3:**
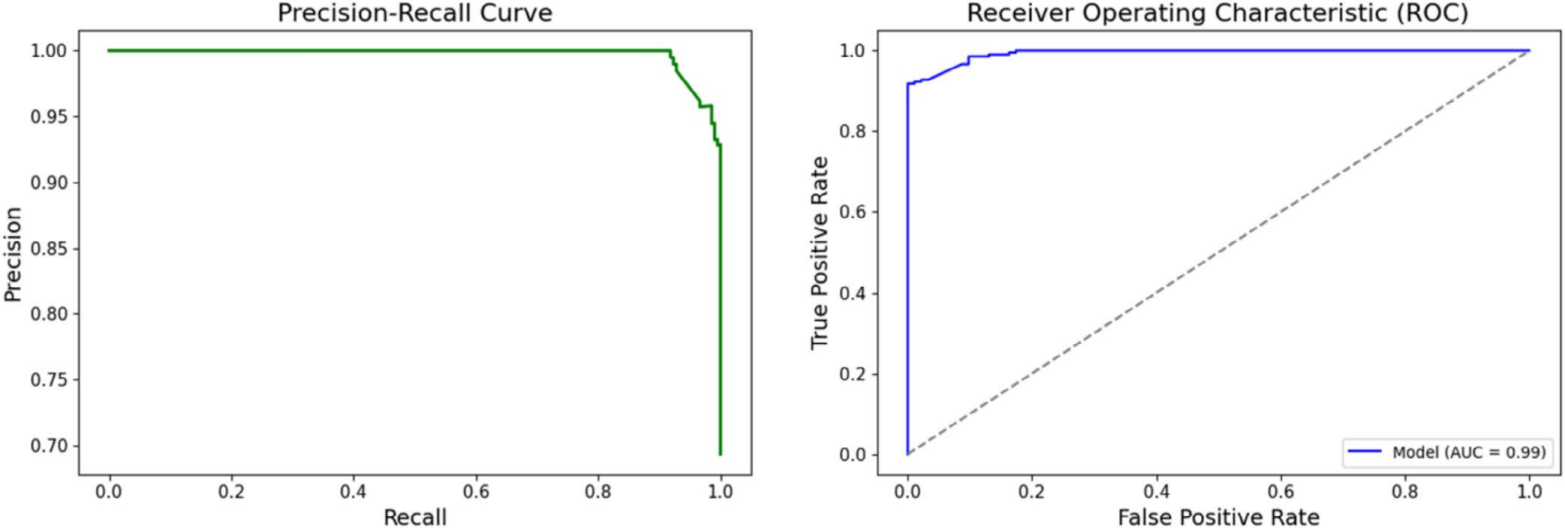
Receiver operating characteristic (ROC) and precision‐recall curve results on the classification task. The ROC curve and precision‐recall curves for the classification task, demonstrating the model's sensitivity and specificity in identifying lung cancer in unseen patient data.

Using the tuned ResNet (encoder) parameters, we extracted features from CT scan images for the classification task. Since the model was initially trained to reconstruct lesion regions, we assumed that the encoded features would likely contain most of the information needed to distinguish between cancerous and normal chest CT scan images. The training process was conducted using the binary cross‐entropy loss function, with accuracy chosen as our monitoring metric (Figure 2). In this setting, the model could achieve an accuracy of 96% on validation data and 94% on the real patient testing data. To prevent overfitting and maintain the model's robustness for other datasets, we tested L2 regularization, weight decay, and various hyperparameters. Ultimately, we found that incorporating a dropout layer with a rate of 0.3 provided more stable training progress, as shown in Figure 2. The results suggest that the model is capable of transferring the knowledge it acquired during U‐Net training to the image classification task. The receiver operating characteristic (ROC) curve results on real patient test data show the robustness of the model on new datasets (Figure 3).

To identify the optimal initialization parameters and model architecture for the encoder, we conducted experiments using various image classification models. The primary variation across these models lay in the encoder, while the decoder followed the reverse architecture of each respective encoder. ResNet was selected as the preferred architecture, as its feature extraction capabilities, when applied to the encoder, consistently outperformed the other models (Table 1).

**TABLE 1:** Comparison of model accuracies for the classification task.

Model	Accuracy
VGG19	92.1
Xception	93.0
InceptionV3	94.4
Densenet121	91.4
EfficientNetB0	94.6

The performance of the conventional ML classification models on the independent test set was evaluated (Table 2). Even though the classical models could achieve high accuracy on the training and validation sets, they consistently did random classification on the independent test set.

**TABLE 2:** Evaluation results using classical machine learning models for the classification task.

Model	Accuracy	Cohen kappa score	Matthews correlation coefficient (MCC)	*F*1 score	Evaluation set
SVM	0.99	0.98	0.98	0.99	Training
XGBoost classifier	0.99	0.97	0.97	0.98	Training
NBC	0.83	0.59	0.60	0.71	Training
KNN	0.99	0.99	0.99	0.99	Training
SVM	0.99	0.99	0.99	0.99	Validation
XGBoost classifier	1	1	1	1	Validation
NBC	0.81	0.56	0.57	0.69	Validation
KNN	0.99	0.99	0.99	0.99	Validation
SVM	0.51	0	0	0	External test
XGBoost classifier	0.51	0	0	0	External test
NBC	0.51	0	0	0	External test
KNN	0.51	0	0	0	External test

## 4 Discussion

This study aimed to develop a neural network model using transfer learning to detect nodules and lesion regions and classify lung CT scans as either cancerous or normal. The primary objective of this paper was not to improve lung cancer classification, but rather to develop a robust model to assist physicians in detecting lung nodules and tumors. To further demonstrate the accuracy of our segmentation, we also trained a classifier using the segmented data. The results indicate that the fine‐tuned ResNet50 model, designed with a U‐Net‐like architecture, successfully detected lung nodules and lesions. This could help the classifier achieve an accuracy of 94% in distinguishing cancerous from noncancerous CT images in a real‐world patient testing dataset. The model showed that it can generalize the task on the independent test dataset, which could not be achieved by classical ML models.

Accurate and early detection of cancers, particularly lung cancer, is critical for improving survival rates and guiding treatment decisions [15, 16]. AI‐derived models play a pivotal role in enhancing diagnostic efficiency and creating comprehensive assessments of available data [17]. Our model's ability to accurately reconstruct tumor regions from CT scans has significant clinical implications. By localizing lesions and nodules, it can assist radiologists in reducing the probability of missed diagnoses, especially for small nodules and in challenging regions of the lung.

In terms of performance, ResNet50 has been widely used in medical imaging due to its robust feature extraction capabilities. A study comparing ResNet50 with other architectures, such as VGG19 and InceptionV3, found that ResNet consistently achieved higher accuracy than VGG19 and performed similarly to InceptionV3 [18]. This aligns with our findings, where ResNet50 achieved an accuracy of 94% for classifying lung cancer. VGG19, a simpler model, is often outperformed by ResNet due to its deeper layers, which lack the residual connections that help ResNet avoid issues like vanishing gradients. VGG19 was shown to have an accuracy of 92.1% in the comparison, lower than ResNet50.

InceptionV3, known for handling varying spatial scales within images, is also competitive in medical imaging tasks. However, in our comparison, InceptionV3 achieved an accuracy of 92.4%, slightly lower than ResNet50, suggesting that while InceptionV3 is effective, ResNet's residual learning framework provides a slight edge in extracting deep features from CT scan data. Interestingly, EfficientNetB0 slightly outperformed both ResNet and InceptionV3 in the study, achieving the highest accuracy of 94.6%, which may be due to its efficient scaling of network width, depth, and resolution. Additionally, CNNs utilize gradient descent (GD); another model reported in the literature has also demonstrated superior performance for lung cancer detection, likely attributed to its custom design specifically tailored for the task [18]. These findings emphasize that model performance can vary depending on the architecture, dataset characteristics, and hyperparameter tuning.

Our findings are consistent with previous research in AI‐based lung cancer diagnosis. For instance, Ardila et al. reported an AUC of 0.94 for detecting lung cancer using CT scans, aligning with the performance of our model [19]. Both studies demonstrate the high potential of deep learning in accurately detecting lung malignancies. Similarly, Schwyzer et al. employed FDG‐PET imaging combined with deep learning for lung cancer detection, reporting a sensitivity of 95.9% and specificity of 98.1% [20]. Our model, using CT scans, achieves comparable sensitivity, further supporting the robustness of deep learning in this domain.

Additionally, the segmentation task in our study aligns with the work of Shen et al., who used a multiscale CNN for lung nodule classification, reporting an accuracy of 88.84% [21]. Our model provides an added advantage by improving the interpretability of the diagnostic process, facilitating more precise localization of tumor regions. Moreover, Feng et al. reported an AUC of 0.912 for distinguishing between invasive and non‐invasive lung adenocarcinoma [22]. In contrast, we achieved a classification accuracy of 94%, driven by the features extracted during tumor segmentation, which were processed through an MLP model.

The classification task, built upon the trained ResNet‐based U‐Net, further validates the robustness of transfer learning in medical imaging tasks. The encoded features from the segmentation task enabled the MLP classifier to achieve high accuracy in identifying lung cancer, demonstrating the efficiency of leveraging pretrained models for feature extraction. This approach highlights the advantages of task‐specific tuning to improve performance across multiple related tasks in medical image analysis.

Recent similar papers have achieved high accuracies in the lung cancer classification task using intensive hyperparameter optimization [23], multimodal training [24], and hybrid features [25]. Although the primary aim of the current paper was not to define a classifier, but rather to implement a U‐net to assist physicians in better tumor diagnosis in hospitals, we were still able to achieve very close accuracy to that reported in the mentioned high‐accuracy papers. Future studies are needed to compare the performance of existing models and conduct a fair benchmarking.

Despite these promising results, several limitations of this study should be acknowledged. Although the model performed well on real patient data, the relatively small size of the test set limits the ability to generalize these findings to larger patient populations. The limited access to larger datasets and the difficulty in obtaining clinical data hinder the progress of this paper; addressing these issues should be prioritized in future studies. Additionally, this study relied on retrospective datasets rather than data from ongoing clinical studies, which does not fully capture the complexities and uncertainties present in real‐world clinical environments. To better validate the clinical applicability and robustness of the model, it would be ideal to conduct simultaneous clinical studies on patients with lung cancer. This approach would allow for real‐time data collection, more diverse patient samples, and continuous monitoring of the model's performance in a true clinical setting. Including more diverse CT scans from different demographic groups and conducting prospective clinical trials will be essential for validating the model's generalizability and efficacy in real‐world medical practice. One possible approach would be to define a reinforcement learning pipeline to improve the performance of the AI model on new data with the help of a professional agent. Furthermore, another area for improvement is the integration of additional data types. While this study focused on image‐based features, future models could incorporate clinical and genomic data, further enhancing patient‐specific diagnoses and treatment decisions.

Another important consideration for clinical translation is model interpretability. For AI tools to be integrated into routine diagnostic workflows, clinicians must be able to understand and trust how decisions are made. In this study, while we focused on achieving high classification accuracy and lesion localization, we acknowledge the need for incorporating explainability techniques in future iterations of our model. In addition to the methodological and clinical considerations discussed above, the ethical and regulatory aspects of deploying AI in healthcare must also be addressed. The integration of AI tools into clinical practice is subject to stringent regulatory requirements. Regulatory frameworks such as the FDA in the United States, EMA in Europe, and data protection laws like HIPAA and GDPR govern the use of AI in healthcare. Although this study is at a research stage and has not yet entered clinical deployment, we acknowledge the importance of adhering to these guidelines in future development phases.

## 5 Conclusion

In conclusion, this study presents a deep learning framework that integrates transfer learning for effective segmentation and classification of lung cancer in CT scans. Unlike traditional ML models, which demonstrate poor generalizability on external datasets, our ResNet50‐based U‐Net architecture, followed by a MLP classifier, achieved high accuracy and consistent performance across multiple evaluation metrics, including *F*1 score, Dice index, MCC, and Cohen's kappa.

The primary goal of this work was to assist clinicians in the early and accurate identification of lung nodules through explainable AI tools. While the model shows strong potential for real‐world clinical application, further prospective validation and subgroup analysis are necessary to ensure robustness across diverse patient populations and imaging environments. Future directions will focus on enhancing model interpretability (e.g., using Grad‐CAM or SHAP), integrating multimodal clinical and genomic data, and exploring regulatory pathways for clinical implementation. By bridging the gap between research and practice, this approach has the potential to support early diagnosis, improve treatment planning, and ultimately enhance patient outcomes in lung cancer care.

## Author Contributions

**Taghi Riahi:** conceptualization, investigation, supervision, project administration. **Bahareh Shateri‐Amiri:** conceptualization, investigation, supervision, project administration, writing – review and editing. **Amirhossein Hajialiasgary Najafabadi:** methodology, software, formal analysis, investigation, writing – original draft. **Sina Garazhian:** methodology, software, formal analysis, investigation, writing – original draft. **Hanieh Radkhah:** supervision, project administration. **Diar Zooravar:** writing – original draft, writing – review and editing. **Sahar Mansouri:** data curation. **Roya Aghazadeh:** data curation. **Mohammadreza Bordbar:** data curation. **Shirin Raiszadeh:** data curation.

## Ethics Statement

In our study, all patient data used from Hazrat Rasool Hospital was fully anonymized prior to analysis, in compliance with institutional ethical standards. Personally identifiable information was removed, and data access was restricted to authorized research personnel only.

## Conflicts of Interest

The authors declare no conflicts of interest.

## Supporting information


**Supporting Information 1 and Figures S1–S6.** Tumor region recognition performance. The model’s effectiveness in reconstructing tumor regions from CT scans, showcasing all test samples with predicted tumor locations compared to ground truth annotations.

## Data Availability

The data that supports the findings of this study are available in Supporting Information of this article.
