# Supplementary figures and images for: Lung Cancer Management: Revolutionizing Patient Outcomes Through Machine Learning and Artificial Intelligence
Source: Cancer Rep (Hoboken). 2025 Jul 17;8(7):e70240. doi: 10.1002/cnr2.70240 (PMC12269930; doi:10.1002/cnr2.70240)

## Supplementary materials

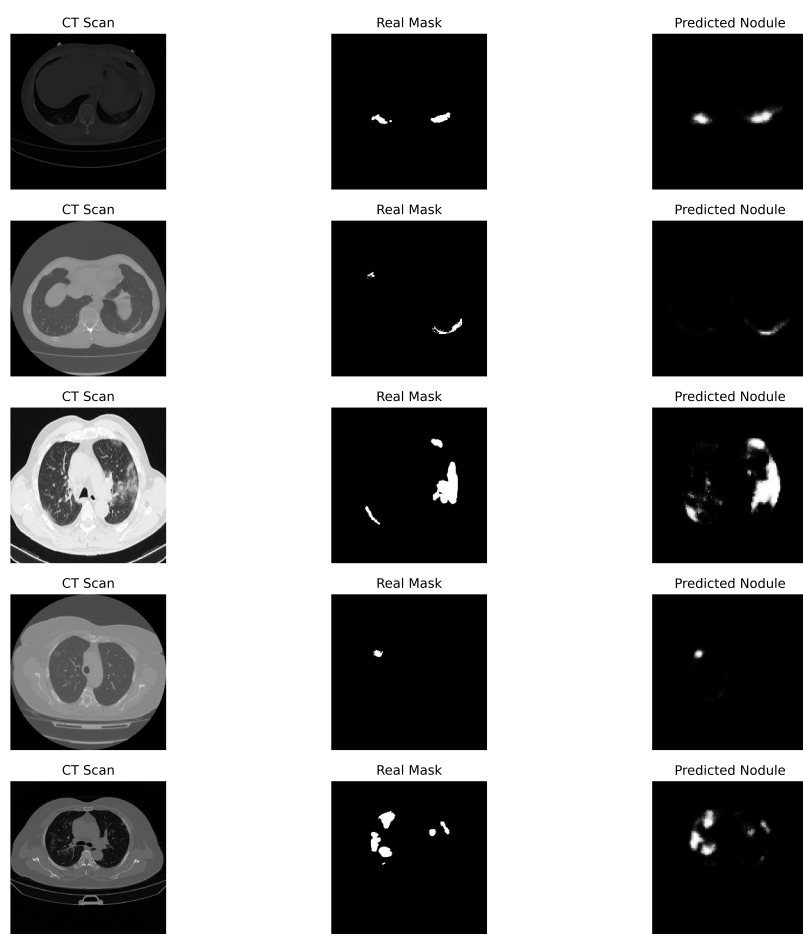

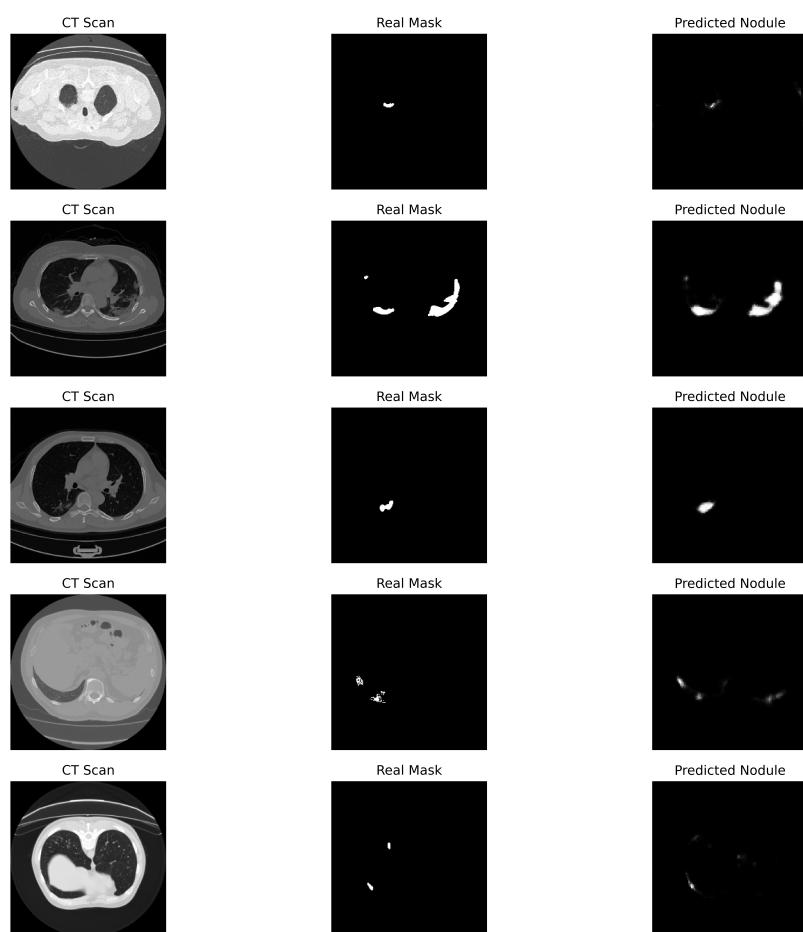

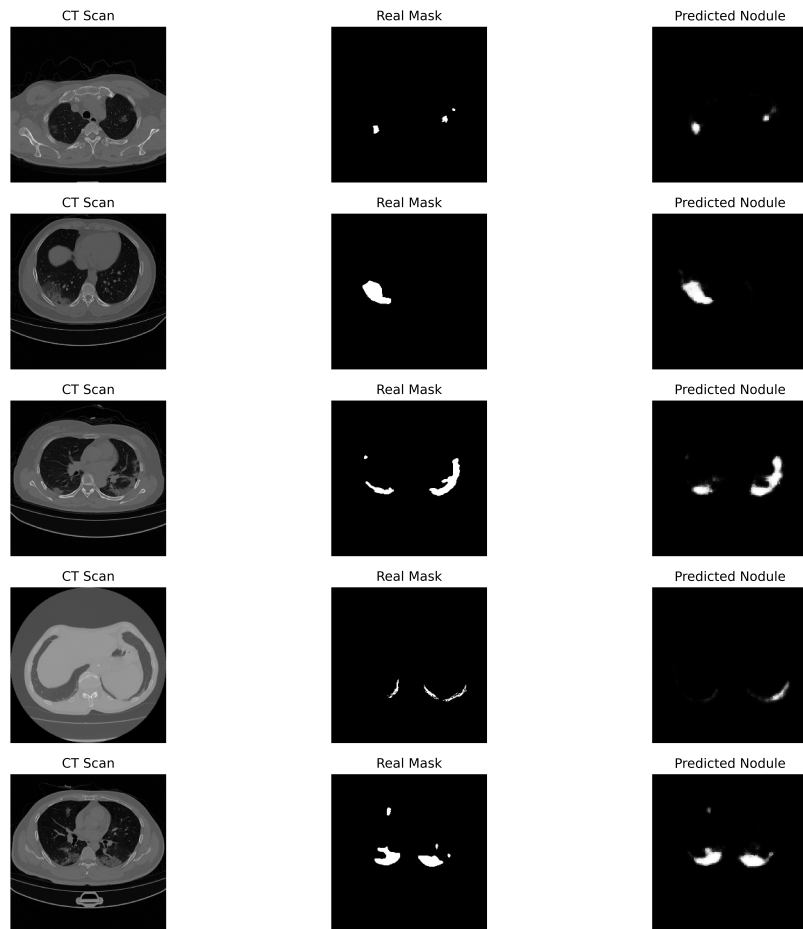

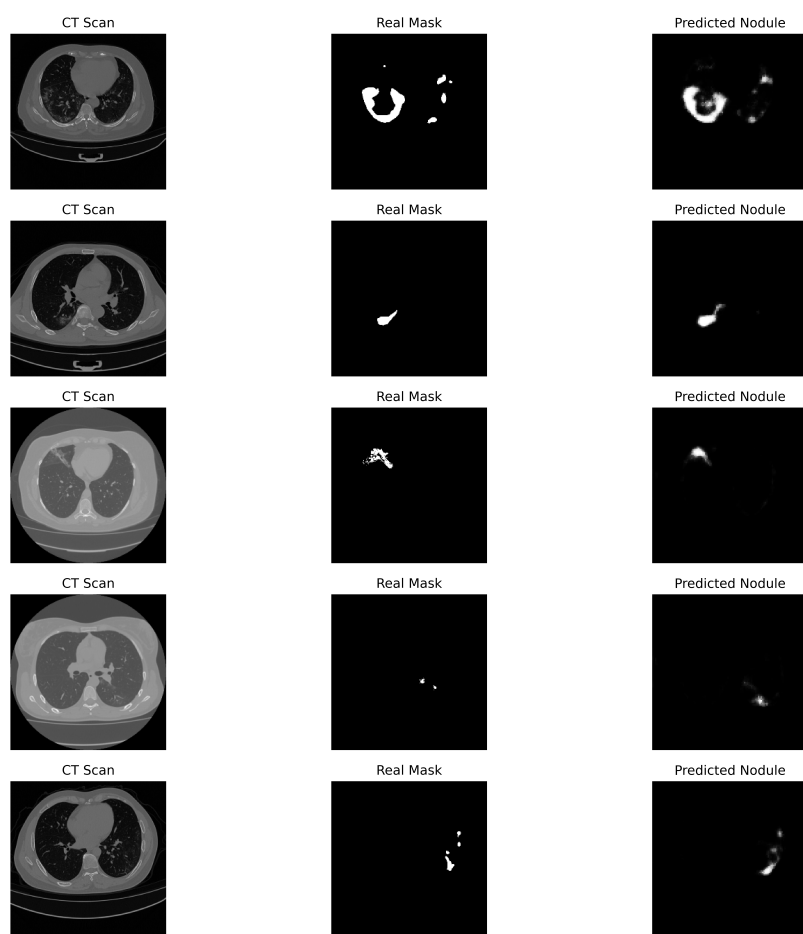

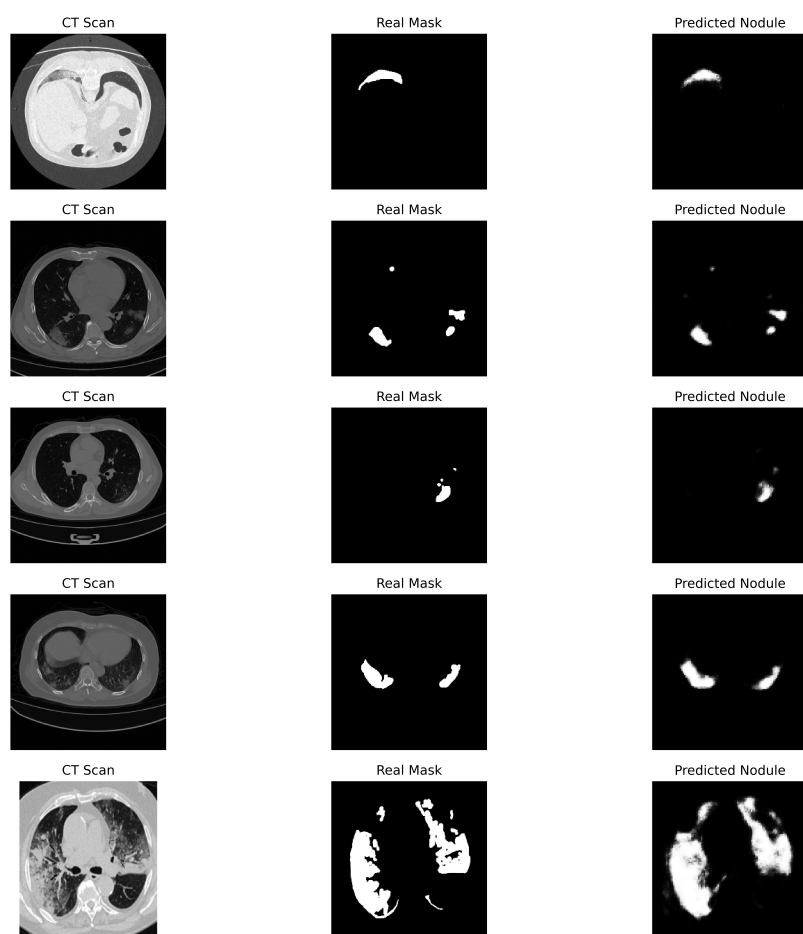

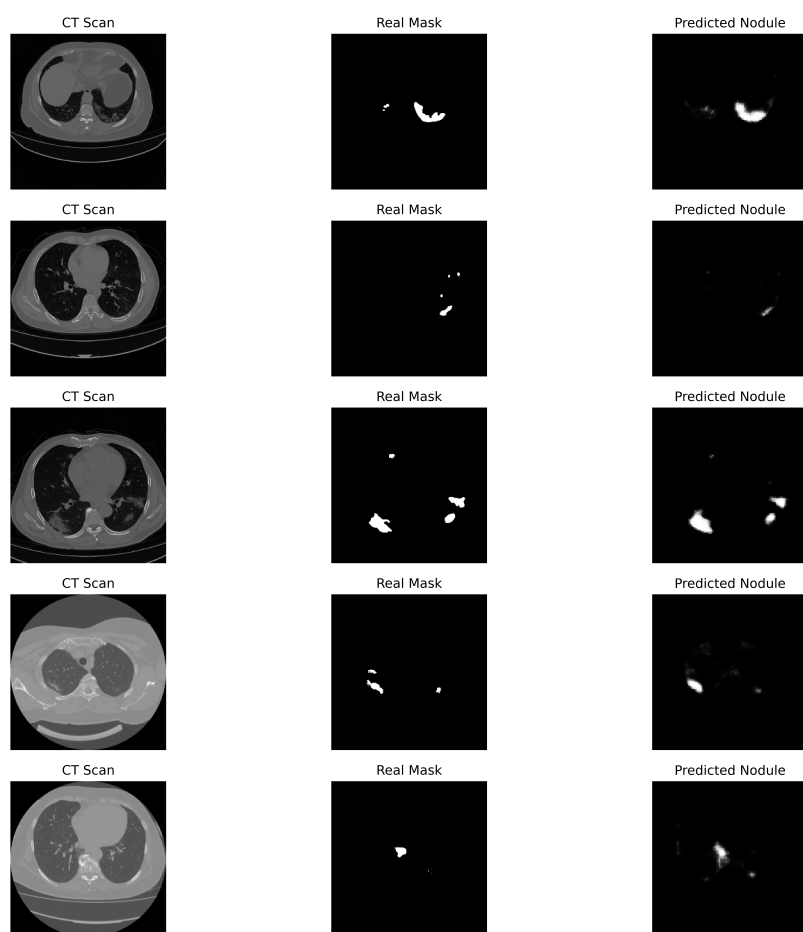

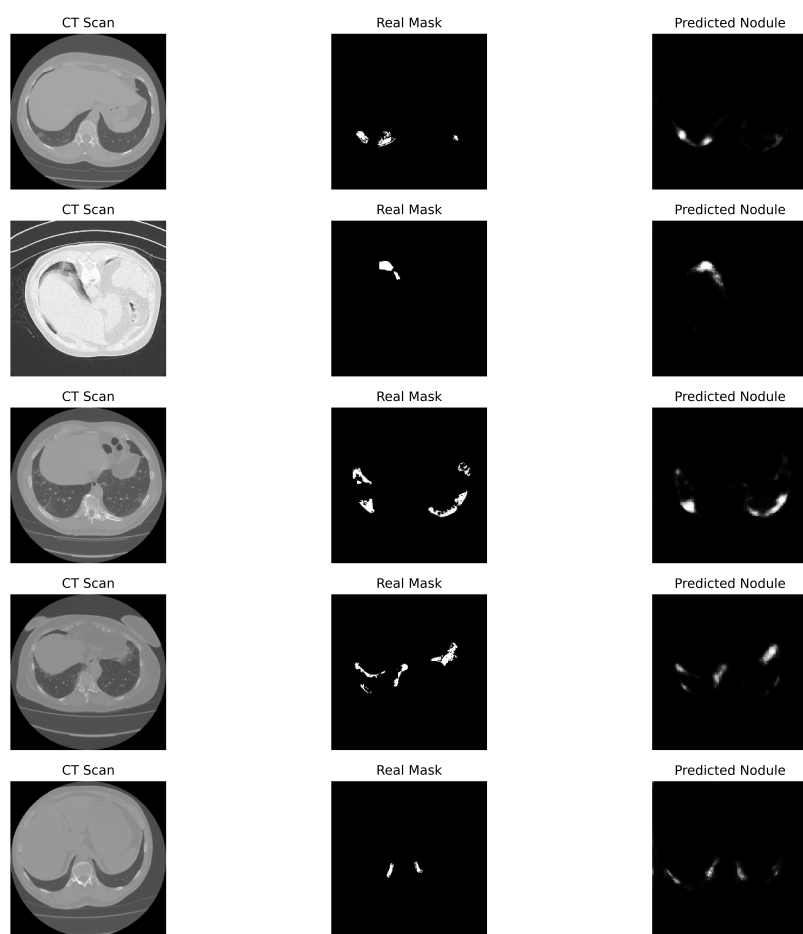

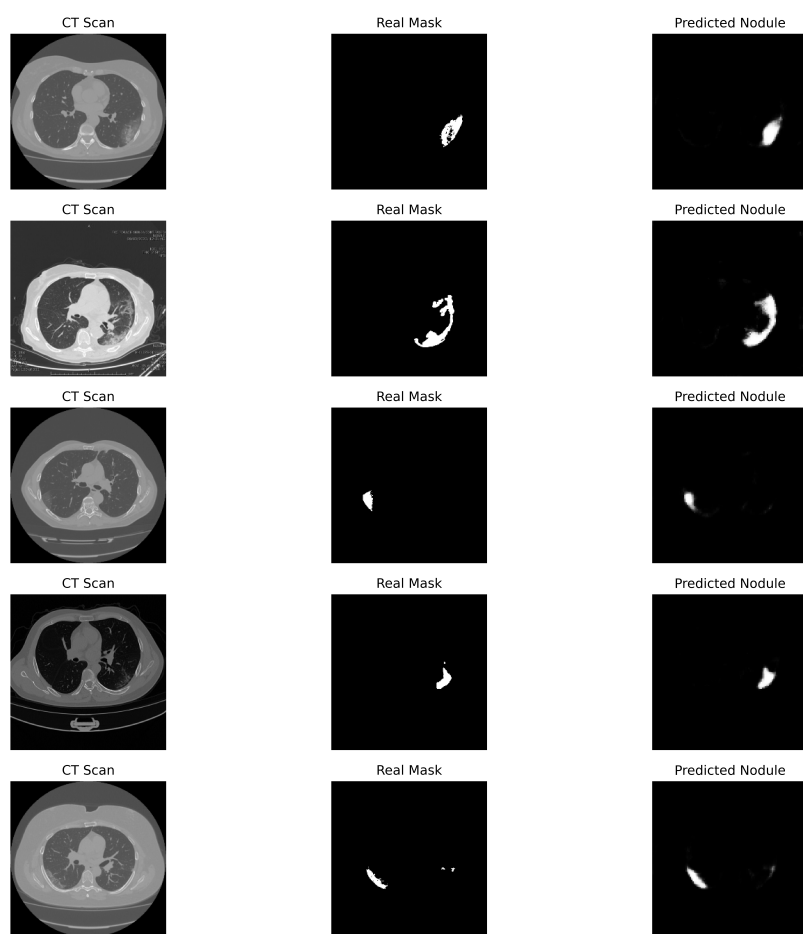

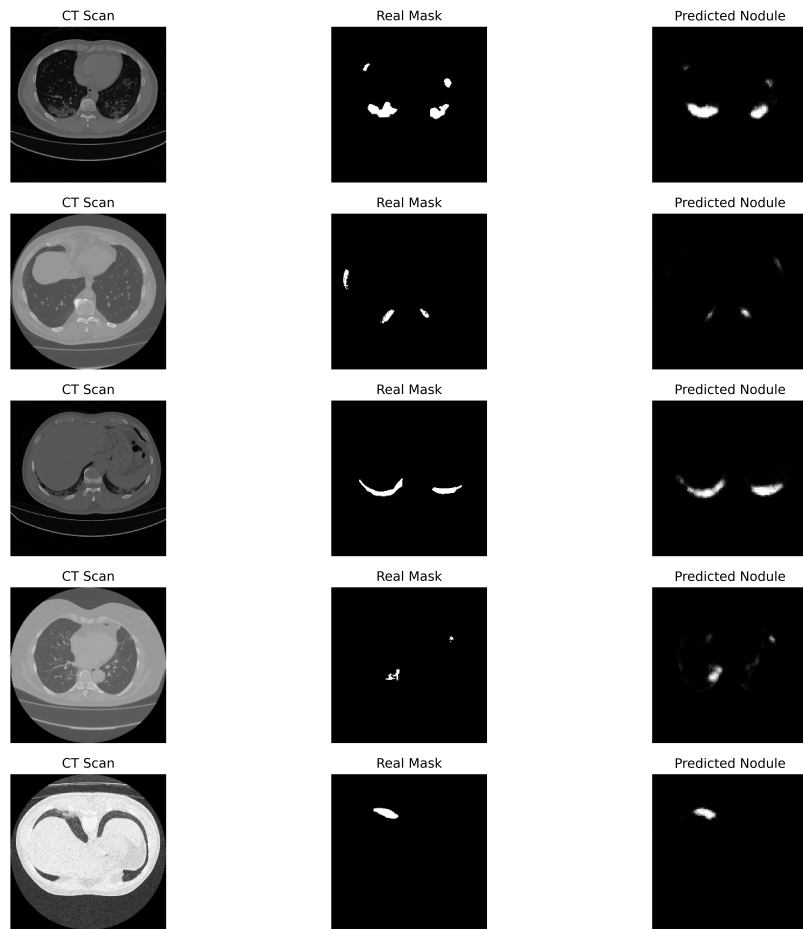

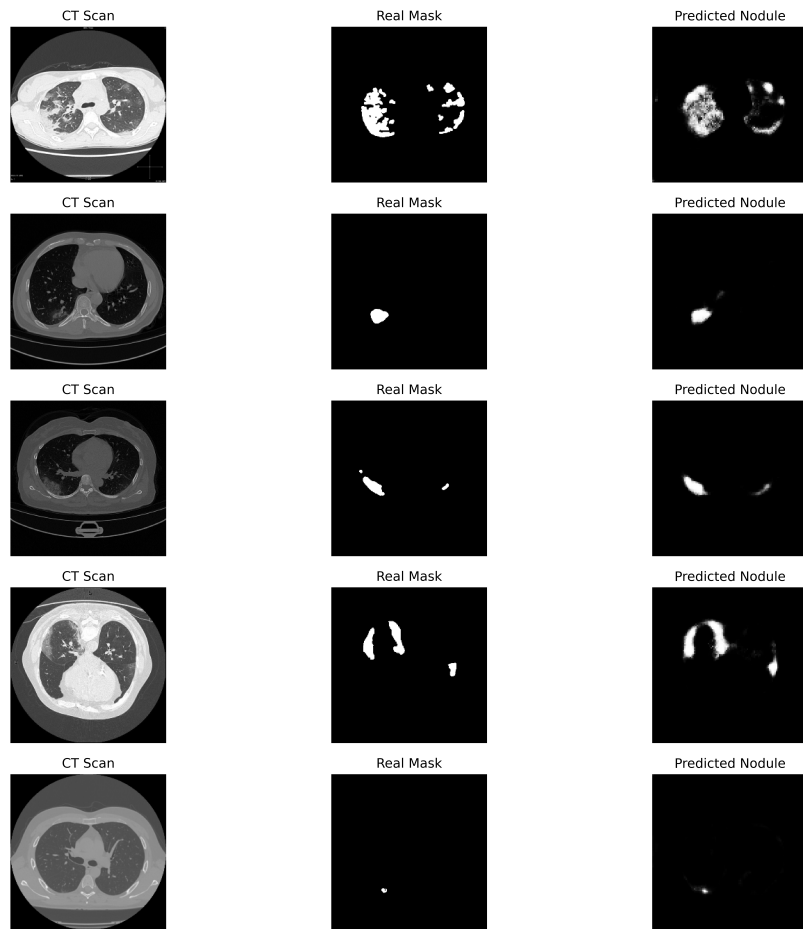

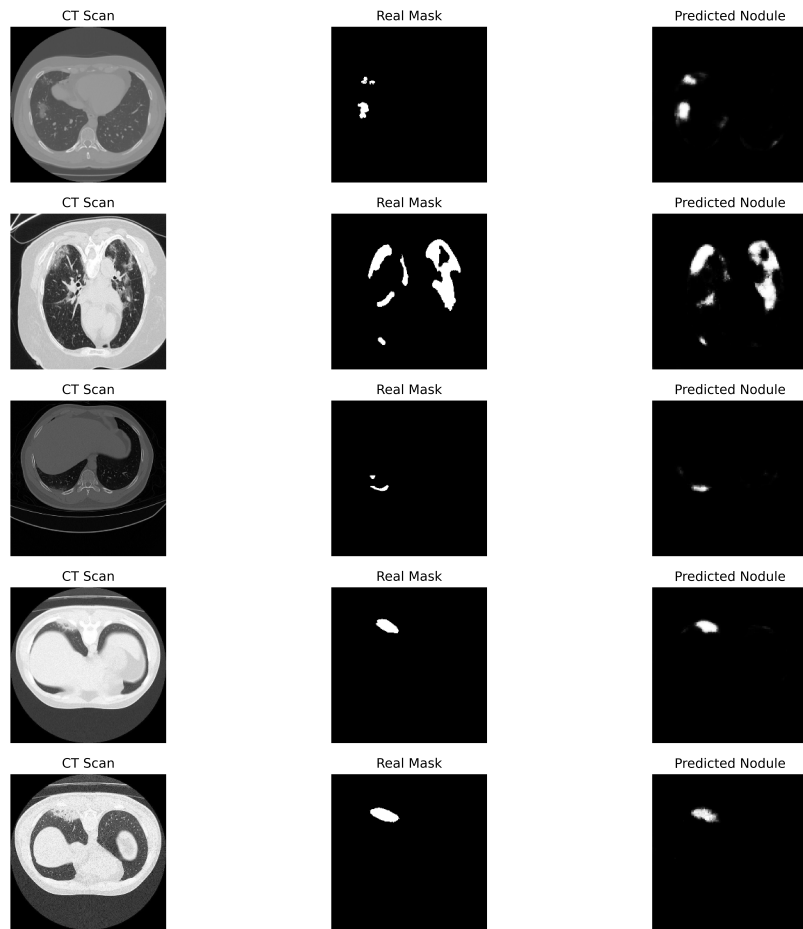

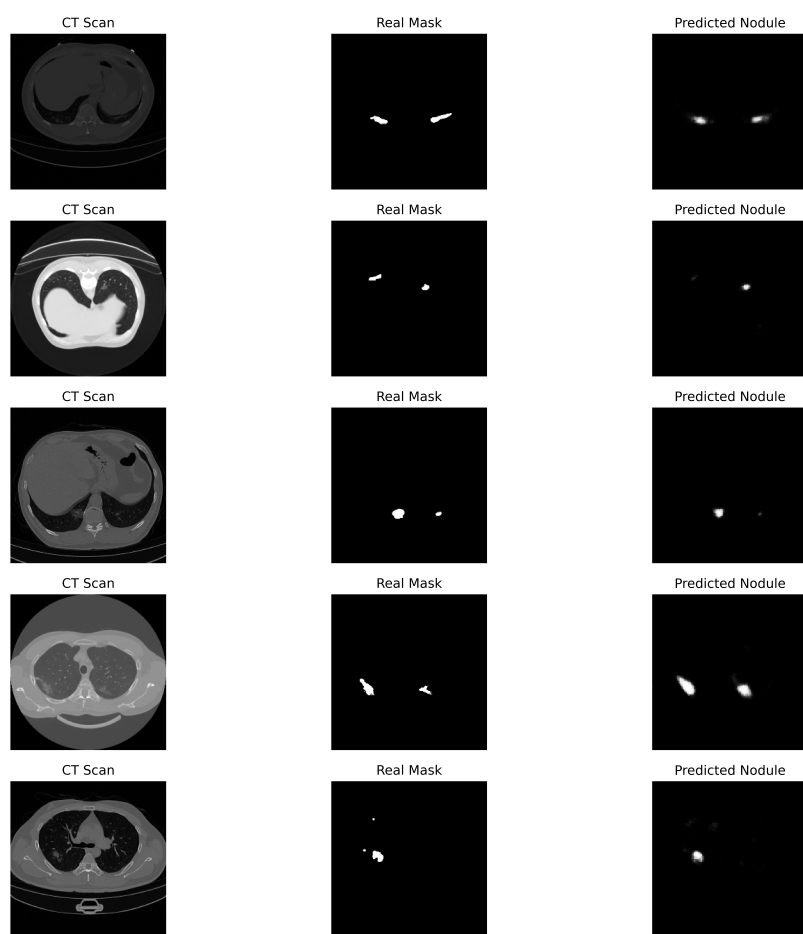

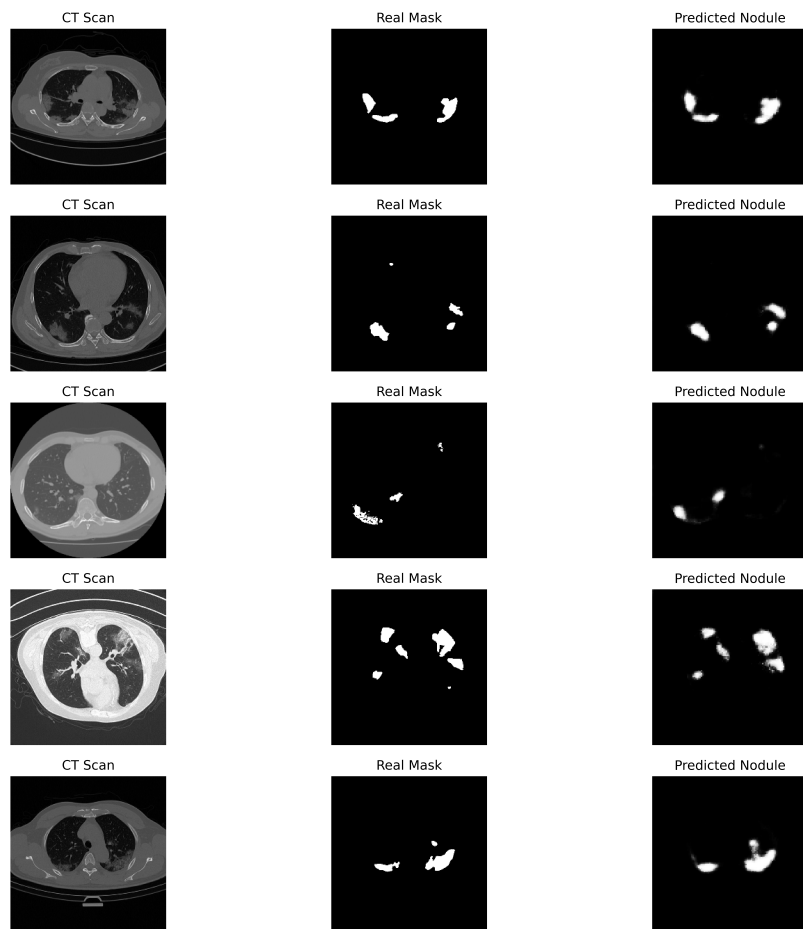

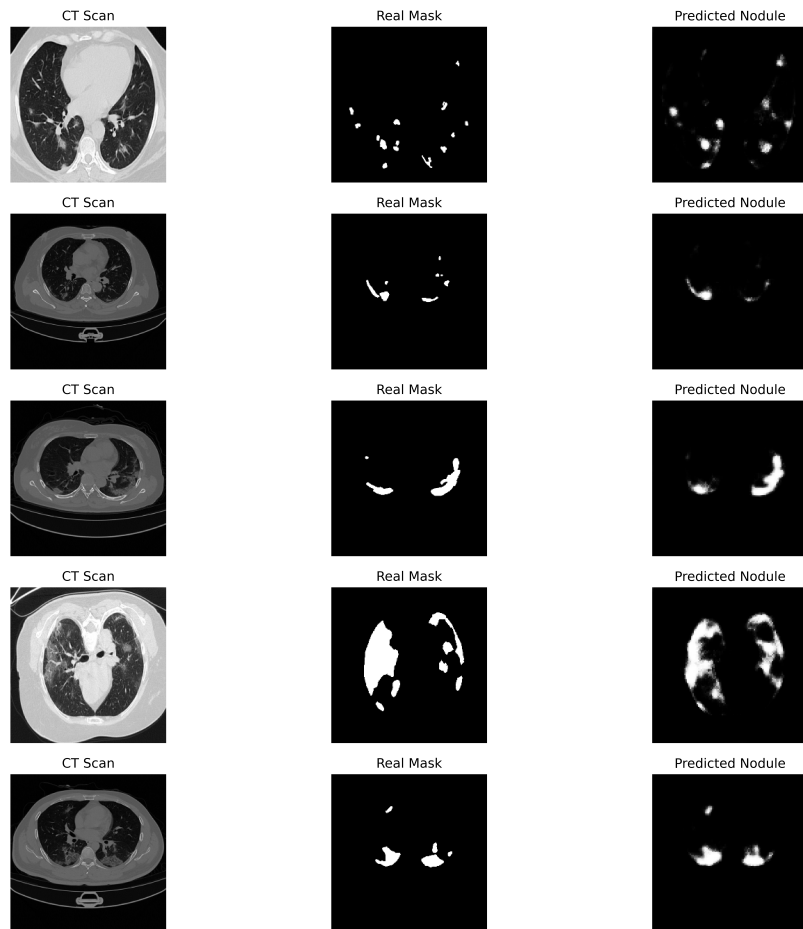

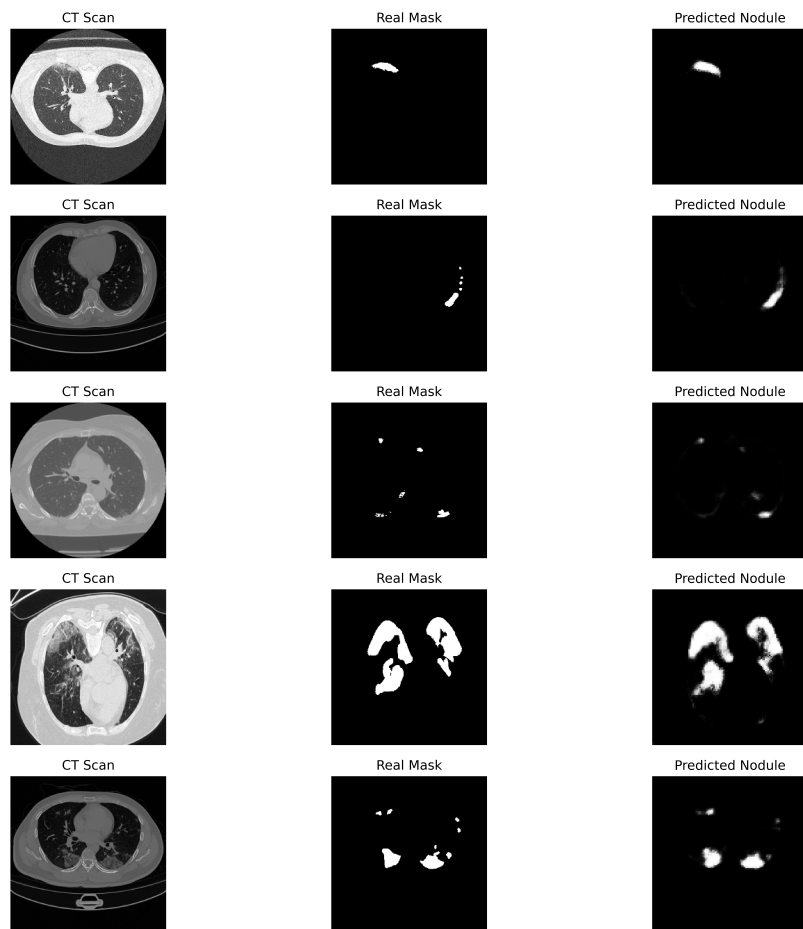

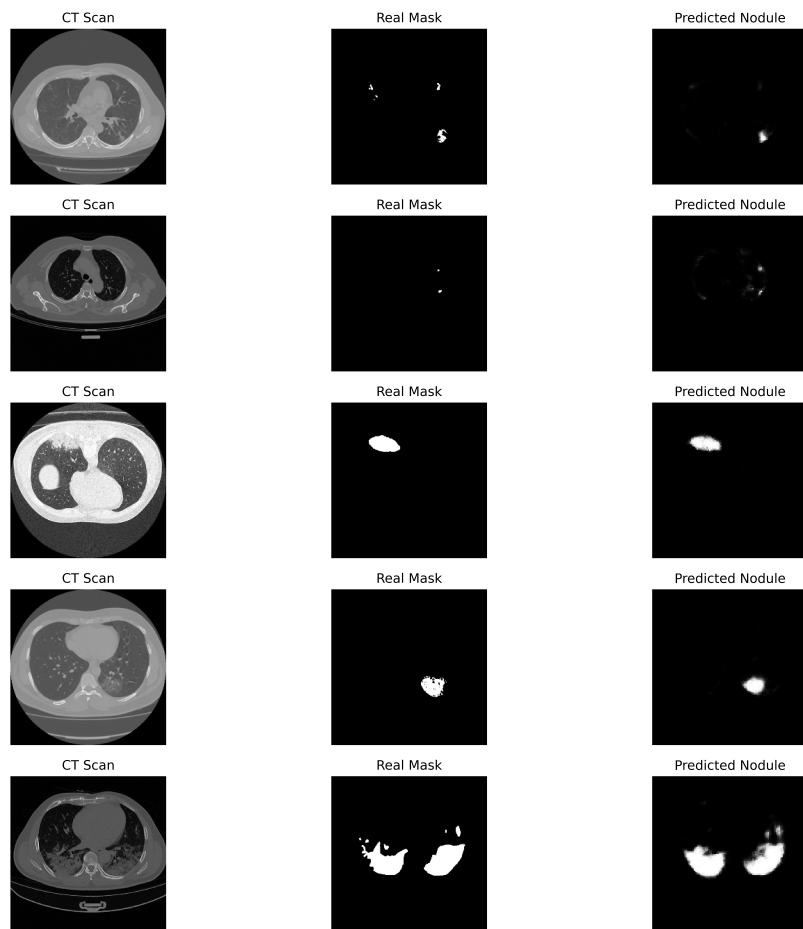

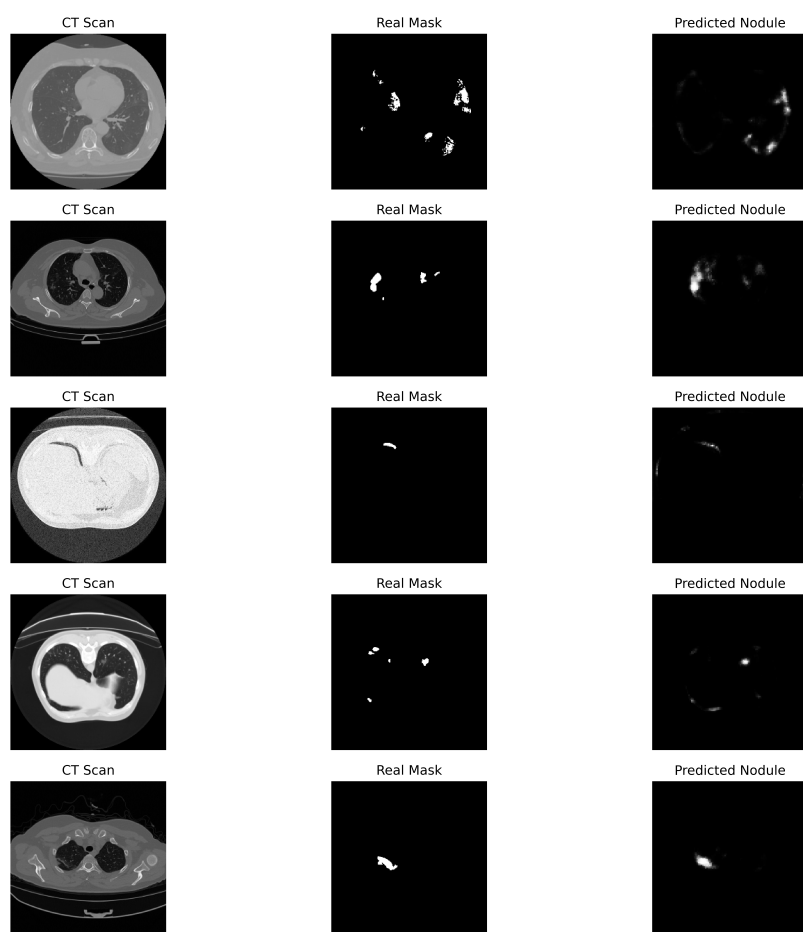

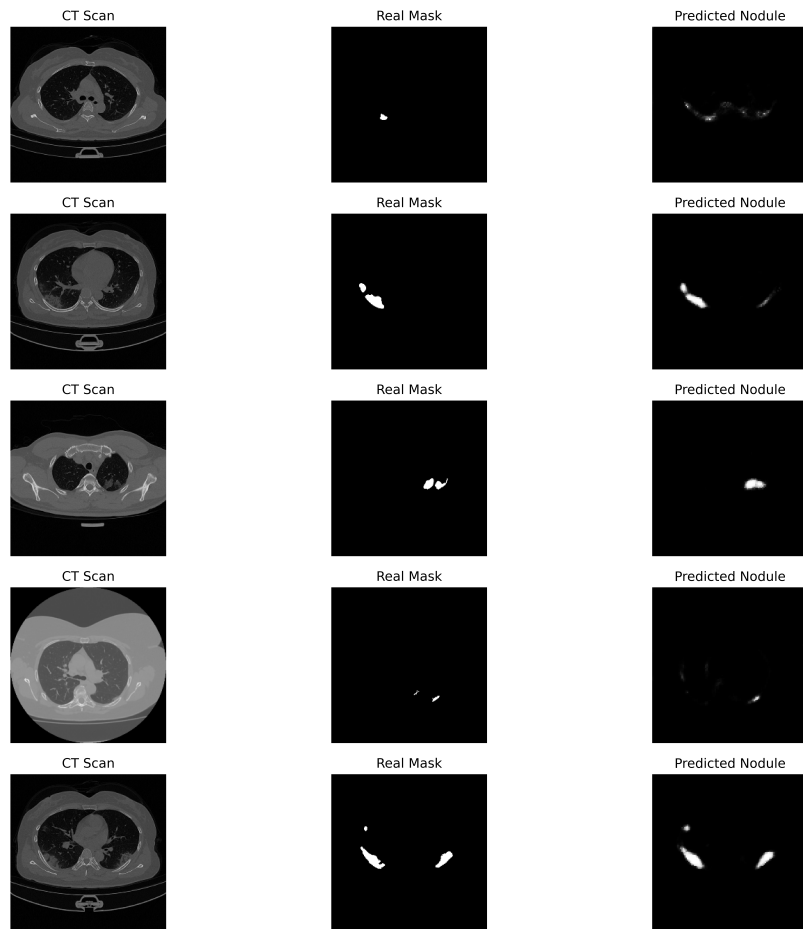

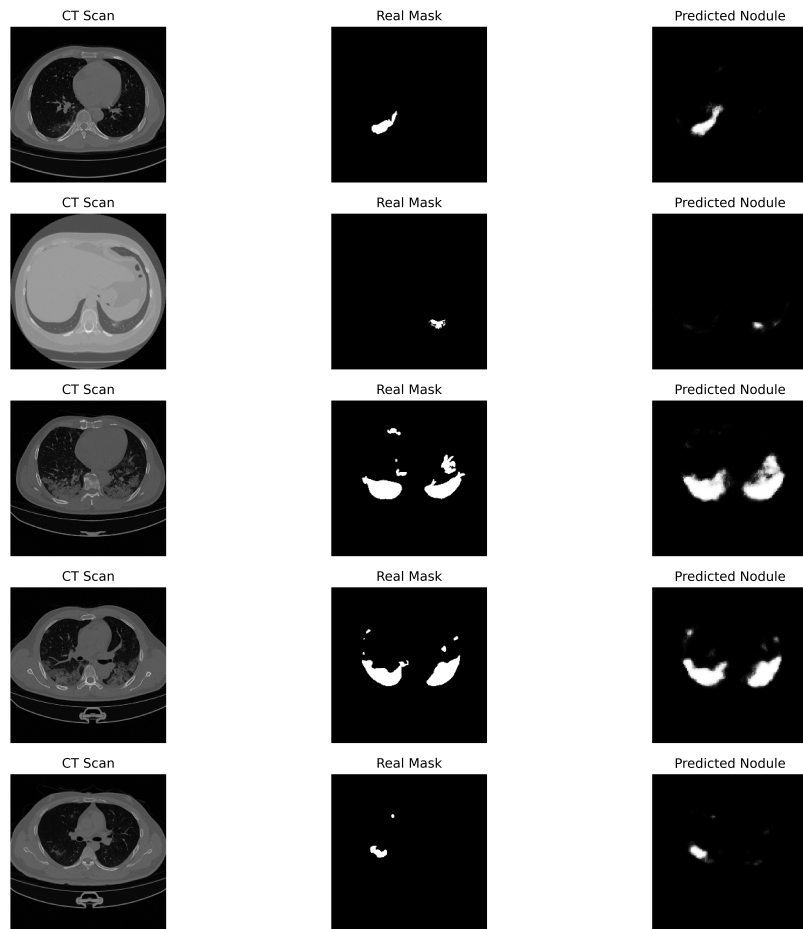

Supplement: Supplementary file 1 — Supporting Information 1 and Figures S1–S6. Tumor region recognition performance. The model’s effectiveness in reconstructing tumor regions from CT scans, showcasing all test samples with predicted tumor locations compared to ground truth annotations. [file CNR2-8-e70240-s001.pdf]
